# The impact of *Anopheles gambiae* egg storage for mass rearing and production success

**DOI:** 10.1186/s12936-019-2691-4

**Published:** 2019-02-26

**Authors:** Ernest Mazigo, Winifrida Kidima, Joseph Myamba, Eliningaya J. Kweka

**Affiliations:** 1 Department of Zoology and Wildlife Conservation, College of Natural and Applied Sciences, University of Dar es Salaam, Dar es Salaam, Tanzania; 2 National Institute for Medical Research, Amani Medical Research Centre, P.O. Box 81, Muheza, Tanzania; 3 Department of Medical Parasitology, School of Medicine, Catholic University of Health and Allied Sciences-Bugando, P.O. Box 1464, Mwanza, Tanzania; 4 Division of Livestock and Human Disease Vector Control, Tropical Pesticides Research Institute, P.O.Box 3024, Arusha, Tanzania

**Keywords:** Mass rearing, *Anopheles gambiae*, *Plasmodium falciparum*, Sterile insect technique, Hatch rate index, Life history traits

## Abstract

**Background:**

Mass rearing requires a large colony from which male individuals can be harvested for sterilization and release. Attention is needed when monitoring life parameters of the reared population, knowing that any variations within the target population would lead to mismatching between two populations. The aim of this study was to assess the impact of *Anopheles gambiae* sensu stricto (s.s.) egg storage on hatchability and life history traits. For each parameter, comparison was made between freshly laid and stored eggs in three densities (40, 80, 120 eggs).

**Methods:**

*Anopheles gambiae* s.s. freshly laid eggs were collected from the Tropical Pesticide Research Institute (TPRI) insectary. Eggs to be stored were kept at − 20 °C for 10 min and then transferred to refrigerators at 4 °C for intervals of 5, 10, 15, 20, and 25 days. After respective storage days, the eggs were transferred from refrigerators to ambient temperature of (25 ± 2) °C for 24 h and then placed in incubators for 24 h. Thereafter eggs were hatched. The egg hatchability, emerged larvae development, larvae survival and emerged adult sex ratios were monitored.

**Results:**

This study found that hatching rates decreased with increase in storage time. The difference was significant in eggs stored for 10 and 15 days (*P* < 0.05). There were no significant differences in hatching rates between *An. gambiae* eggs stored for 5 days and freshly hatched eggs (*P* > 0.05). *Anopheles* larvae development (L1 to pupae) was not significantly affected by storage time across all hatching densities. The study also found that larvae survival decreased with increase in egg storage time. However, there was no significant difference between larvae from freshly hatched eggs and those from eggs at 5 and 10 storage days (*P* > 0.05) but not for eggs stored for 15 days. Furthermore, there was a decrease in emerged adult males and increase in females relative to increased time of egg storage. The difference was significant (*P* < 0.05) at 15 storage days but not for eggs stored for 5 and 10 days (in triplicate densities).

**Conclusion:**

From this study it was concluded that storing *An. gambiae* eggs at 4 °C and 48 ± 2% relative humidity (RH) for 5 days is the optimal condition and time that did not affect egg hatching rates, larval development and survivorship and emerged adult mosquito sex ratio.

## Background

*Anopheles gambiae* is a group comprised of eight members that are morphologically indistinguishable sibling species of mosquitoes in the genus *Anopheles* namely *Anopheles melas, Anopheles merus, Anopheles arabiensis, Anopheles bwambwae, Anopheles coluzzii*, *Anopheles quadriannulatus*, *Anopheles ahimaricus* and *Anopheles gambiae* sensu stricto (s.s.) [1]. Among these, *An. gambiae* s.s.*, An. coluzzii* and *An. arabiensis* are the most efficient malaria vectors in the sub-Saharan region [1, 2]. Malaria prevalence is high, keeping at risk more than 3.2 billion worldwide, 92% in the sub-Saharan Africa region where 10 countries have high malaria transmission based on the 2018 World Health Organization (WHO) malaria report [3]. However, malaria vector population decline has been reported from several parts of Africa where transmission was recorded to be higher before installation of various intervention tools implementation [4–7].

The use of long-lasting insecticide-treated nets (LLINs), indoor residual spraying (IRS), improved diagnosis, and treatment with artemisinin-based combination therapy (ACT) has contributed to the decrease in malaria burden in endemic areas [7, 8]. For instance, the 2016 global malaria burden report presented a worldwide decline of mortality rates from 2010 by 38%, with a 42% decrease reported in Africa [9]. The data show promising trends towards the WHO strategy for elimination of malaria by 2015–2030 [10]. Unfortunately intensive use of insecticides has led to the vector reduced susceptibility status in various parts of Africa [11–15]. Apart from insecticide resistance, behavioural changes recorded in some places have been recognised as a type of insecticide resistance [14]. For example, malaria vectors exhibit some behavioural changes to avoid intra-domiciliary control tools [16–18]. Such behavioural changes include a tendency to bite in the early hours of the night or at dawn, which circumvents current control strategies [16, 19]. As proposed by the World Health Malaria Control Programme, elimination of malaria in African endemic areas by 2030 will not be possible if these challenges are not met [9, 10, 20]. There is the necessity to establish alternative vector control strategies: sterile insect technique might be one of them [21]. Sterile insect technique is a biologically based method to control disease vectors through the introduction of sterile males into the target population [22].

Sterile insect technique is a novel intervention requiring mass rearing of insects under laboratory conditions, exposing the males to gamma rays to induce sexual sterility before release into a target population [22]. Mosquito eggs are known to be the only stage at which diapauses to embryo development can be induced by certain environmental conditions, allowing storage for specific intervals of time without affecting egg viability and other life parameters [23]. Mass rearing of mosquitoes requires large number of eggs from which an experimental population would be harvested [24]. Studies on viability of *Anopheles* eggs showed that eggs hatch within 2 days post-oviposition under optimal environmental conditions (27 °C ± 2, 80% ± 10 relative humidity (RH) [25, 26]. However, changes in environmental conditions have been shown to affect egg viability and induce delayed hatching in mosquitoes [27]. In the laboratory, *Anopheles* eggs can be preserved in different conditions with some resultant certainties of their viability, developmental parameters and emerged adult sex ratios (females emerged against total mosquitoes (males and females emerged)) [23, 28]. There is a need to establish specified laboratory conditions under which eggs would be handled and stored for optimal breeding output of mosquitoes with traits similar to that of the wild population for effective insect sterile technique. This study aims at investigating the impact of laboratory storage conditions, particularly temperature, on hatching rates of *An. gambiae* eggs and associated life history traits.

## Methods

### Study area and study design

The study was conducted in the Livestock and Human Diseases Vectors Division, Tropical Pesticide Research Institute (TPRI), Arusha. The institution insectaries have *An. gambiae* s.s. Kisumu strain (R 70) established since 1992 with a colony from Kisumu, Kenya. The insectary is equipped with necessary instruments for managing and rearing mosquitoes. Such instruments include humidifiers, electric heaters, 12:12 h synchronized lighting schedule, thermometers and hygrometers.

*Anopheles gambiae* freshly laid eggs were collected from the TPRI insectary. A total of 30 filter papers, each with more than 480 eggs, were used for the complete study. A single experiment used 5 filter papers (with more than 2400 eggs). A total of 6 experiments were carried out in triplicate densities (40, 80 and 120 eggs). A total of 14,400 eggs were used for the complete study (= 30 hatching filter papers).

For a single experiment 5 hatching filter papers were randomized into five groups of storage days: the first filter paper was stored for 5 days, the second for 10, the third for 15, the fourth for 20, and the fifth for 25 days. Each filter paper (with about 480 eggs), was divided into two parts containing equal number of eggs (n = 240): one portion was used as control and the other (n = 240) for experiment. The control (n = 240) was further sub-divided into three different densities (n = 40, n = 80, n = 120). Each was then hatched separately into hatching plates of 15 × 10 × 8 cm in size with 500 ml of tap water placed under standard conditions of temperature (27 ± 1) °C and relative humidity (80 ± 10) % [29]. The experimental filter papers (with 240 eggs) were stored following procedures described below.

### Procedures for storing *Anopheles gambiae* eggs

Each filter paper containing 240 eggs was placed separately on top of wet cotton wool in different 100 × 15 mm petri dishes. For a single experiment, a total of 5 petri dishes each with 240 enclosed eggs was used. The 5 petri dishes were covered with a cap, tightened with masking tape and then randomized to different storage days (5, 10, 15, 20, and 25 days). The petri dishes were then placed into deep freezers at − 20 °C for 10 min to heat-shock and stop embryo development before being transferred into refrigerators for storage at 4 °C and 48 ± 2% RH. Petri dishes were then removed from the refrigerators after respective required storage days. After refrigeration, the petri dishes were immediately placed at ambient temperature (25 ± 2) °C, and 78 ± 2% RH for 24 h. Thereafter, the petri dishes were transferred into an incubator at (27 ± 2) °C, (90 ± 10) % RH for another 24 h. After this, filter papers with eggs were removed from petri dishes and eggs were counted manually using hand lens and counter machine. The eggs were placed into hatching plates of 15 × 10 × 8 cm in size, filled with 500 ml of tap water in triplicate egg densities of 40, 80 and 120. Eggs were hatched at 27 ± 1 °C and 80 ± 10% RH. The experiments were repeated six times.

### Impact of *Anopheles gambiae* egg storage time on hatching rates

Hatching rates were monitored twice per day (08:00 and 18.00 h) from when the eggs were washed from filter paper to rearing plate after 24 h of incubation. For each single experiment, the record of eggs hatched was obtained by counting first-instar larvae (F1) within recipient hatching plates. The total F1 in recipient plates was considered equal to the number of viable eggs per density.

### Impact of *Anopheles gambiae* egg storage time on larval development

Emerged larvae were daily fed with Tetramin fish food (Tetra GmbH, Herrenteich 78, 49324 Melle, expiring March 2019) at the rate of 0.0003 gm per larval [30]. Rearing temperature and humidity were regulated and maintained at 28 ± 2 °C and 78 ± 2% RH (Humidifiers, electric heaters and probe type T, Digitron Thermometer Instruments were used) [29].

Mosquito larvae size and larval developmental were used to monitor variations in larval development. Larvae developmental records were taken twice per day (08:00 and 18:00) after which the days on which all larvae in respective densities pupated or emerged into adults were analysed. Dead larvae were recorded and removed from plates using plastic pipettes. Pupae were put into paper cups (70 cm internal diameter with 100 ml of dichlorinated water) and transferred to rearing cage (30 × 30 × 30 cm) for adult emergence, at 27 ± 2 °C and 78 ± 2% RH. Larval development was recorded according to hatching densities and storage duration. Larval developmental time at different hatching densities and storage days were estimated as the average duration from first-instar larval hatching to emergence of adult mosquitoes.

### Impact of *Anopheles gambiae* egg storage time on larvae survivorship

Live and dead larvae were counted, after which the dead were removed from rearing plates. Survival rates of *An. gambiae* larvae were calculated as proportional ratio of daily larval count at a given stage to the total hatched eggs of that density. Proportion computations were separated into densities and egg storage days.

### Impact of *Anopheles gambiae* egg storage time on adult sex ratio

Emerged adults were fed with 10% sucrose sugar, removed from cages using mouth aspirator at age 6–7 days post-emergence. Antennae and mouth parts were used to sort them into sex. Adult sex ratios were computed as the number of emerged adult females divided by the total number of emerged adults (males and females). Data were recorded according to egg hatching density and egg storage days.

### Statistical analyses

Data obtained were cleaned for layouts using MS-Excel. Analysis was done by using Graph Pad Prism software version 7.04 (Graph Pad-Prism Software, Inc. Oxford University 2017). Data were tested for normality using Shapiro–Wilk test. Study results were descriptively summarized using frequencies, proportions, means and standard deviations. Daily hatch computations were done for every 24 h. Hatch records were separated into triplicate densities (40, 80 and 120 eggs) and storage time. Comparison of hatching rates, larval development and sex ratios between stored and freshly laid eggs were analysed using paired samples t-test. The Kaplan–Meier survival analysis was done to determine the effect of storage time on larval survivorship proportions against freshly hatched eggs in similar densities. Adult sex ratios and larval survivorship were calculated by proportional tests. Associated charts and graphs were done using Microsoft Excel 2007 (Microsoft, WA, USA).

## Results

### Impact of egg storage time on hatching rates of *Anopheles gambiae*

**Fig. 1:**
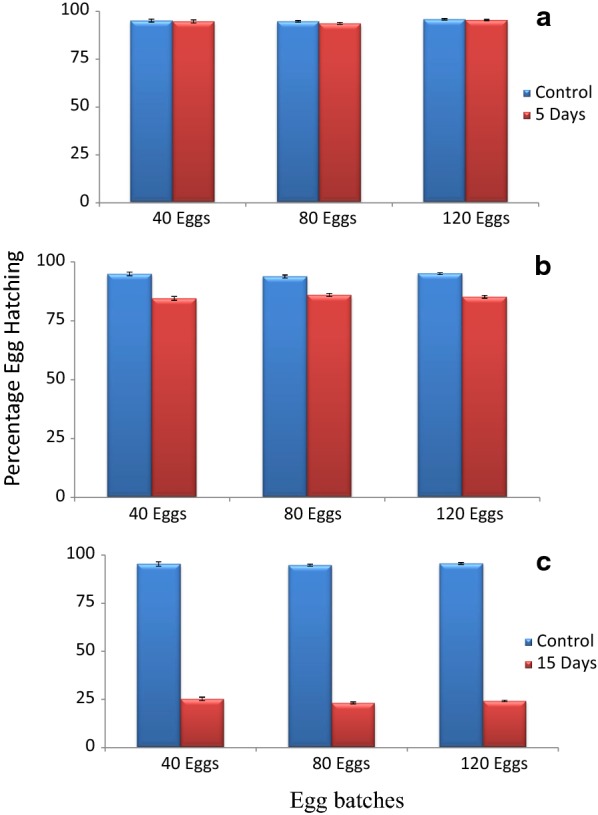
Mean hatching rates of *Anopheles gambiae* s.s. stored eggs. **a** 5 days, **b** 10 days, **c** 15 days and freshly laid eggs. Asterisk indicates a significant difference in hatch between freshly laid and stored eggs by paired sample T-test *P < *0.01* (**P < 0.001; ***P < *0.001*)

Higher mean hatch rates were observed on eggs stored for 5 days. There was an abrupt decrease in hatching rates for eggs stored for 10 and 15 days (Fig. 1b, c). About 93% of eggs stored for 5 days at densities of 40, 80 and 120 hatched successfully. Hatching rates between eggs stored for 5 days and freshly laid eggs at 40, 80 and 120 densities were not significantly different (Fig. 1a, Table 1). Percentage hatch rates of eggs stored for 10 days at 40, 80 and 120 densities were above 84% although they were significantly lower than hatching rates of freshly laid eggs at similar densities (Fig. 1b, Table 1). Eggs stored for 15 days had hatching rates below 29% and significantly lower than those for freshly laid eggs (Fig. 1c, Table 1). Eggs stored for 20 and 25 days did not hatch.

**Table 1:** of the mean hatching rates between freshly hatched and stored *An. gambiae* s.s. eggs at different densities

Density	Freshly hatched and stored eggs hatched at different densities		
	5 days	10 days	15 days
40 eggs	t = 0.31, *P* = *0.77*	t = 8.73, *P* < *0.01*	t = 62.61, *P* < *0.001*
80 eggs	t = 2.07, P = 0.09	t = 11.35, *P* < *0.01*	t = 76.32, *P* < *0. 001*
120 eggs	t = 0.72, P = 0.49	t = 14.69, *P* < *0.01*	t = 250.32, *P* = *0. 001*

### Larval development between similar hatching densities of freshly laid and stored *Anopheles gambiae* eggs

Development time increased with increase in storage time. Larvae from eggs stored for 15 days took more days to pupate than those from days 5 and 10. Overall larvae from stored eggs took longer to pupate than those from freshly laid eggs although the difference was not significant at 5, 10 and 15 storage days in the triplicate densities (Table 2).

**Table 2:** The effects of storage of *An. gambiae* s.s. eggs on larval development time from larval to adult stage

Eggs storage period	Hatching density (n)	Larval developmental time (Mean ± SD) (days)				
		Duration L1 to pupal			Duration L1 to adults	
		Freshly laid	Stored eggs	P values (L1 to pupal stage)	Freshly laid	Stored eggs
5 days	40	7.1 ± 1.31	7.7 ± 1.49	t = 1.981, P = 0.12	10.2 ± 1.49	10.6 ± 0.52
	80	7.9 ± 1.71	8.3 ± 1.79	t = 2.409, P = 0.12	12.8 ± 1.79	12.0 ± 0.63
	120	8.7 ± 1.91	9.3 ± 2.11	t = 1.942, P = 0.12	13.4 ± 2.11	13.7 ± 0.81
10 days	40	7.2 ± 1.35	7.9 ± 1.54	t = 1.689, P = 0.17	10.9 ± 1.54	10.8 ± 0.41
	80	8.0 ± 1.72	8.7 ± 1.81	t = 2.361, P = 0.08	12.7 ± 1.83	13.5 ± 0.55
	120	8.8 ± 1.98	9.4 ± 2.23	t = 1.696, P = 0.17	13.9 ± 2.23	14.3 ± 0.52
15 days	40	7.3 ± 1.42	8.3 ± 1.89	t = 1.086, P = 0.34	10.3 ± 1.89	10.0 ± 0.63
	80	8.2 ± 1.79	8.9 ± 1.94	t = 0.705, P = 0.52	12.9 ± 1.94	13.1 ± 0.55
	120	8.9 ± 2.09	9.8 ± 2.26	t = 0.991, P = 0.38	13.8 ± 2.26	13.7 ± 0.52

### Impact of *An. gambiae* egg storage time on larval survivorship at different densities

**Fig. 2:**
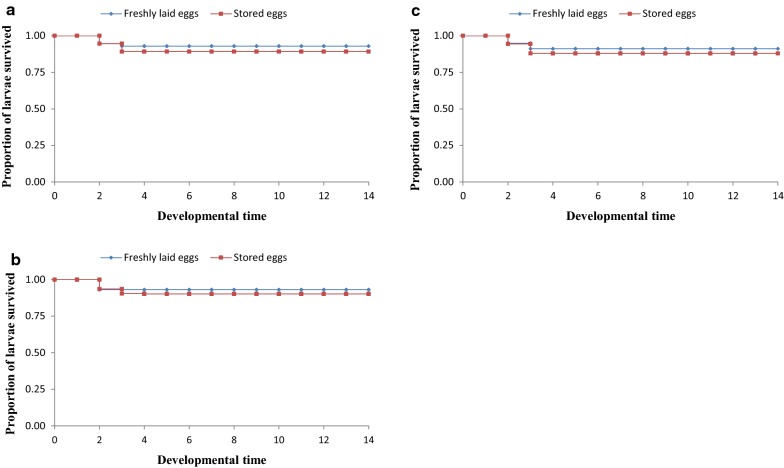
Larval survivorship curves for eggs stored for 5 days at 4 °C, 48 ± 2% RH and larvae from freshly laid *Anopheles gambiae* s.s. eggs at different densities. **a** 40 eggs, **b** 80 eggs, **c** 120 eggs (six experiments in triplicates)

**Fig. 3:**
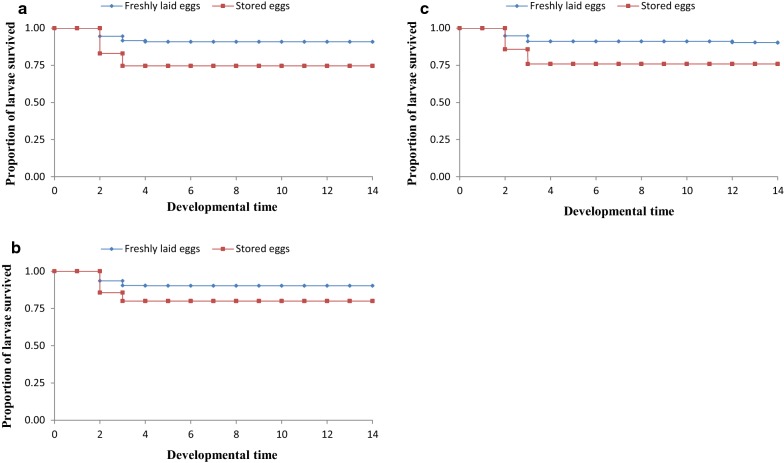
Larval survivorship curves for eggs stored for 10 days at 4 °C, 48 ± 2% RH and larvae from freshly laid *Anopheles gambiae* s.s. eggs at different densities. **a** 40 eggs, **b** 80 eggs, **c** 120 eggs (six experiments in triplicates)

**Fig. 4:**
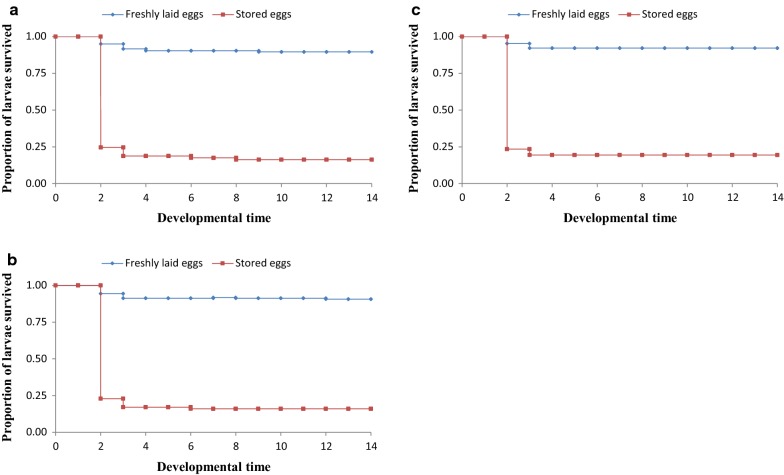
Larval survivorship curves for eggs stored for 15 days at 4 °C, 48 ± 2% RH and larvae from freshly laid *Anopheles gambiae* s.s. eggs at different densities. **a** 40 eggs, **b** 80 eggs, **c** 120 eggs (six experiments in triplicates)

Proportions of larvae surviving to adult stage decreased with increase in egg storage time (Table 3). Survival rates of larvae from both freshly laid (control) and stored eggs (experimental) dropped after day 2 of hatching, with mortality rate being more pronounced in larvae from stored eggs (Figs. 2, 3 and 4). After day 3, the survival rates were almost steady to adult stage. There was no significant difference in proportions of larvae survived to pupal stage between those from freshly laid eggs and stored eggs for 5 days across the three hatching densities [(χ^2^ = 0.223, *P* = 0.637) at 40 eggs, (χ^2^ = 0.208, *P* = 0.648) at 80 eggs, and (χ^2^ = 0.197, *P* = 0.657) at 120 eggs (Fig. 2)]. Variation in larval survivorship was observed in larvae emerged from eggs stored for 10 days, although the difference was not statistically significant across all hatching densities [(χ^2^ = 2.035, *P* = 0.154) 40 eggs, (χ^2^ = 1.407, *P* = 0.236) 80 eggs, and (χ^2^ = 2.3, *P* = 0.129) 120 eggs (Fig. 3)]. However, variation in larval survivorship was observed to be different between larvae emerged from freshly laid eggs and those stored for 15 days [(χ^2^ = 5.249, *P* = 0.022) at 40 eggs, (χ^2^ = 6.211, *P* = 0.013) at 80 eggs, and (χ^2^ = 6.454, *P* = 0.011) at 120 eggs (Fig. 4)].

**Table 3:** Effects of storage of *An. gambiae* s.s. eggs on larval survivorship from larval to adult stage

Eggs storage period	Hatching density (n)	Larval survivorship (%)			
		Survival l1 to pupal		Survival L1 to pupal	
		Freshly laid	Stored eggs	Freshly laid	Stored eggs
5 days	40	92.9 ± 1.49	91.2 ± 1.01	91.5 ± 1.49	91.2 ± 1.01
	80	93.1 ± 0.76	90.6 ± 1.14	91.6 ± 0.76	90.6 ± 1.14
	120	91.1 ± 1.72	89.6 ± 1.32	92.6 ± 1.72	87.6 ± 1.43
10 days	40	91.7 ± 0.82	78.6 ± 1.93	91.7 ± 0.82	77.3 ± 1.04
	80	90.4 ± 0.82	81.6 ± 0.97	90.4 ± 0.82	81.6 ± 0.97
	120	91.1 ± 1.37	79.5 ± 1.53	91.1 ± 1.37	79.5 ± 1.53
15 days	40	91.7 ± 1.03^dx^	18.8 ± 1.34	91.7 ± 1.03^dx^	18.8 ± 1.34
	80	91.5 ± 1.09^dx^	17.2 ± 0.67	91.5 ± 1.09^dx^	17.2 ± 0.67
	120	92.2 ± 1.03^dx^	19.4 ± 1.84	92.2 ± 1.03^dx^	19.4 ± 1.84

^(dx)^Letters superscripts indicates that values in respective rows are significantly different at P < 0.05 by Kaplan–Meier test

### Comparison of sex ratios between adults from freshly laid eggs and those from stored eggs of *Anopheles gambiae*

**Fig. 5:**
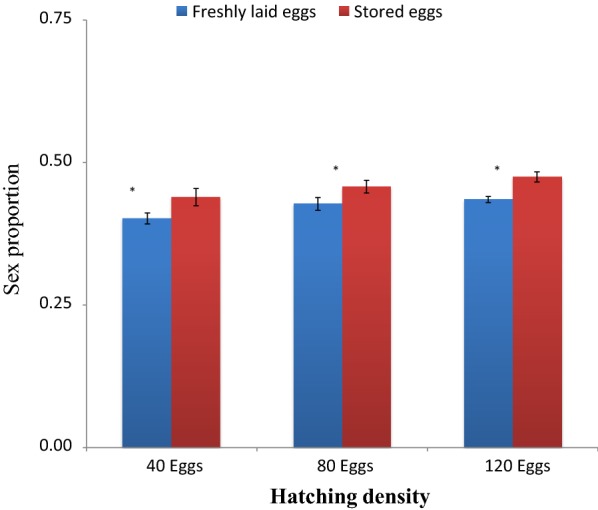
Sex ratio of adult *Anopheles gambiae* s.s. (females emerged against total mosquitoes) (males and females emerged) from freshly laid eggs and eggs stored at 4 °C and 48 ± 2% RH for 5 days and hatched at different densities. Asterisk indicates a significant difference at *P* < *0.05* by paired sampled test. Six experiments in triplicates

**Fig. 6:**
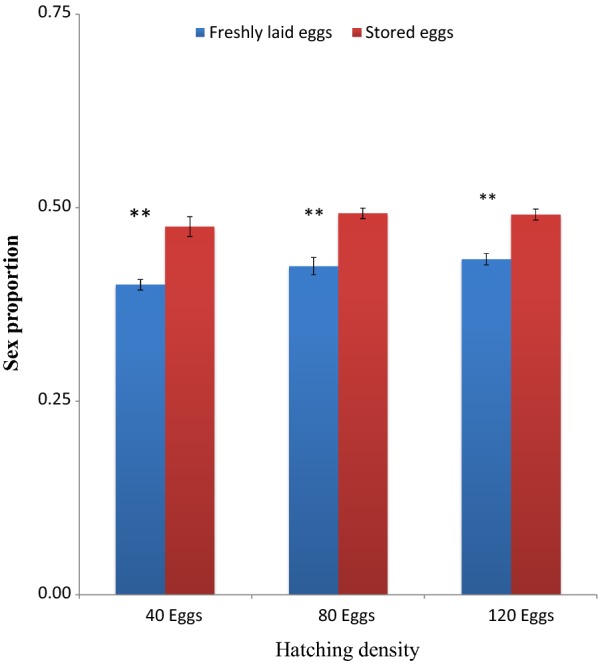
Sex ratio of adult *Anopheles gambiae* s.s. (females emerged against total mosquitoes) (males and females emerged) from freshly laid eggs and eggs stored at 4 °C and 48 ± 2% RH for 10 days and hatched at different densities. Double asterisk indicates a significant difference at *P* < *0.001* by paired sampled test. Six experiments in triplicates

**Fig. 7:**
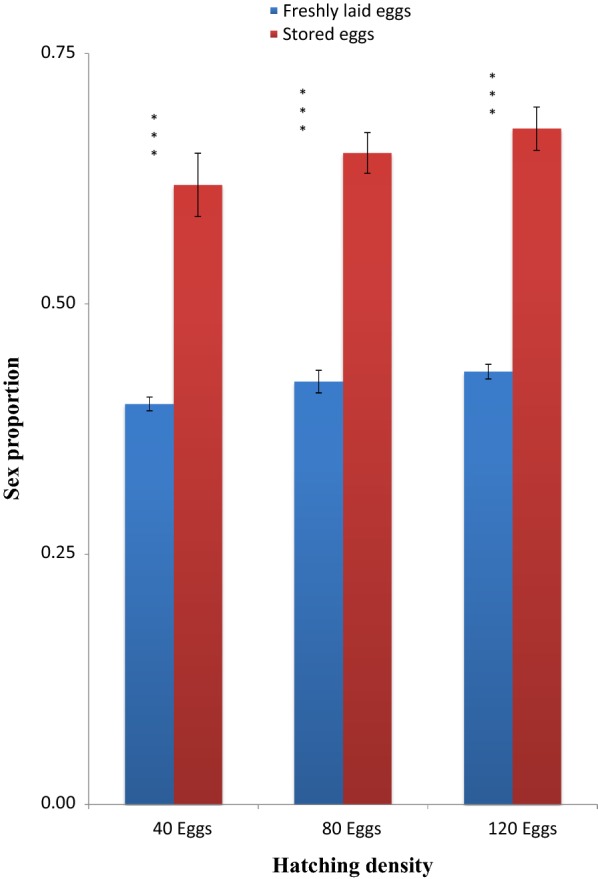
Sex ratio of adult *Anopheles gambiae* s.s. (females emerged against total mosquitoes) (males and females emerged) from freshly laid eggs and eggs stored at 4 °C and 48 ± 2% RH for 15 days and hatched at different densities. Triple asterisk indicates a significant difference at *P* < *0.0001* by paired sampled test. Six experiments in triplicates

The sex ratio of adult *An. gambiae* emerged from freshly hatched eggs across all densities was lower than 0.5, indicating more males than females in the emerged population. The sex ratios of adult mosquitoes emerging from eggs stored for 5 and 10 days were also lower than 0.5. However, sex ratios of adult mosquitoes emerging from eggs stored for 15 days was above 0.5, indicating that there were more females than males. Statistical analysis revealed that sex ratios of adult mosquitoes emerging from eggs stored for 5 days across all densities were statistically higher than those from freshly hatched eggs of similar densities (t = 4.99, *P* = *0.004*) at 40 eggs, (t = 6.69, *P* < *0.001*) at 80 eggs, and (t = 8.23, *P* < 0.001) at 120 eggs densities (Fig. 5). There was significant differences between sex ratios of adult mosquitoes emerging from eggs stored for 10 days and those emerging from freshly hatched eggs at 40 eggs (t = 8.74, *P* < 0.001), at 80 eggs (t = 14.45, *P* ≪ *0.001*), and at 120 eggs (t = 20.90, *P* < *0.001*) (Fig. 6). The same was found in sex ratio of adults emerged from eggs stored for 15 days and freshly hatched eggs (t = 19.08, *P* < *0.001*) at 40 eggs, (t = 19.36, *P* < 0.001) at 80 eggs, and (t = 19.23, *P* < 0.001) at 120 eggs (Fig. 7).

## Discussion

The current study underlays a foundation for *An. gambiae* egg storage effect on egg viability, larvae development and emerged adult sex ratio which is crucial information on the mass rearing of mosquitoes. The aim of the present study was to investigate the effects of storing *An. gambiae* eggs at − 20 °C for 10 min and then transferring to 4 °C and 48 ± 2% RH on hatchability, larval development, larval survivorship, and emerged adult sex ratios. It was found that storage time had negative consequences on egg hatchability, larval development rates and larval survivorship as number of storage days increased. Additionally, there was skewed sex ratio as adult females increased as egg storage time increased.

These findings show that egg hatchability patterns decreased with increased time of egg storage; 93 and 85% hatch rates were recorded at 5 and 10 storage days, respectively. This pattern was similar to studies conducted by Khan and others in which egg hatch rates were reported to decrease with increase of egg storage time at 16 °C [23]. Relatively high hatch rates were also reported in *An. gambiae* that were stored at 12 °C for 1 and 3 days, with reduction in hatch rate observed for eggs stored for longer, for 7 and 10 days [31]. Embryonic development has been recognized to be highly influenced by external stimuli particularly temperature [31]. It has been shown that exposure of mosquito eggs to temperature below the optimal threshold (24 °C) may lead to diapauses [32]. It follows that prolonged exposure in diapause state depletes embryological resources and consequently results in embryo mortality [31]. Brown and others have reported a decrease in hatch rate with an increase in storage time in *Aedes aegypti*, i.e., for every added day of *Aedes aegypti* egg incubation at 26.5 °C, hatch rates dropped by 0.27% [33]. Hatch rate index (HRI) negatively associates with time of egg exposure into diapauses. Since storing eggs for 10 days resulted in significantly lower hatch rate compared to freshly laid eggs in this study, storing eggs for 5 days at 4 °C and 48 ± 2% RH is considered optimal duration as it maintained higher egg hatchability compared to freshly laid eggs.

The effect of egg storage on larval development has shown that *An. gambiae* larval development was inversely proportional to storage time at all densities although the difference was not significant. The findings of this study have been found similar to previous reported a delayed *An. gambiae* and *Aedes aegypti* larval development with increased egg storage time [23, 31]. Reduced locomotor and feeding activities have been reported in diapause-terminated larvae [34]. Several studies have reported a strong shift in gene expression patterns and regulatory pathways during diapauses [35, 36]. The shift in gene expression patterns may lead to lack of normal physiological development. Delayed larval development observed in larvae from stored eggs in the present study might have resulted from impacts of diapauses and changes in gene expression patterns, as previously reported in various studies [23, 31]. The evidence shows that the rate of insect larvae growth and development gradually diminishes as the exposure of micro-embryo to a lower temperature threshold increases [37, 38]. According to Beck-Johnson et al. when insect eggs are subjected to lower temperature thresholds, micro-embryo physiological processes, including development, are retarded or sometimes ceased [39]. Physiological processes are regained when developing embryos are subjected to normal temperature thresholds, although retarded effects have been shown to determine subsequent developmental parameters [40]. In the present study, larvae that emerged from eggs stored for 5 days at 4 °C and 48 ± 2% RH took less time (8.4 days) to pupate than larvae from eggs stored for 10 and 15 days (8.6 and 9 days, respectively). That is, the pupation period increased with increased time of egg storage. Larval development trends observed in this study are likely similar to previous studies on *An. arabiensis* and *Culex quinquefasciatus*, in which larval developmental time increased relatively to increase in storage time [23, 41]. The delayed larval development observed in the present study might have been induced by thermal effects of storage temperature (4 °C, 48 ± 2% RH) and increased time of storage on embryogenesis.

This study revealed that increase in storage time had negative consequences on larval survivorship. This means that larvae mortality was inversely proportional to egg storage time. Higher larval mortalities were recorded in eggs stored for 15 days. In insectary practices it is known that when mosquito eggs are stored in humid conditions, embryos may mature before eggs are washed into hatching plates [42, 43]. Longer exposure periods in diapauses before hatching were likely to affect larval survivorship as food and energy are exhausted before hatch. Similar to this study are findings from Russell and others who observed a decline in *Ae. aegypti* larvae survivorship with an increase in storage time at 25–26 °C and 95% RH [44]. This suggests that storing eggs might have made the larvae too feeble to survive soon after hatch as has been shown in the present study and previous studies [45, 46].

The results from this study have several practical applications. When *An. gambiae* eggs are stored in moistened condition at lower temperatures they can still be utilized for seeding collection. At 4 °C, 48 ± 2% RH, *An. gambiae* eggs may be kept for 5 and 10 days with an anticipated hatch of 93 and 85%, respectively, and with no effect on rate of survivorship of emerging larvae. The ability to store *Anopheles* eggs for a period of time at stated conditions in this study permits flexibility in accommodating holidays or absence of personnel for mass rearing of mosquitoes.

The emerged sex ratio of eggs in this study was found to be affected by storage days. Dipterans larval sexes, particularly mosquitoes, are known to exist in either heterozygous or homozygous of male and female and expression of sexes are known to be thermally affected based on the range of temperature to which developing embryo and larvae are subjected [47, 48]. In natural populations, males outnumbers female mosquitoes [49, 50]. In the present study, the proportions of adult *An. gambiae* females were lower than males in both freshly laid eggs and those eggs stored from 5 to 10 days. Proportions of males to females in this study were skewed relatively to time the eggs were stored. Insect sex determination, particularly in dipterans, are said to be accidental, highly induced by temperature and controlled by sex-specific lethality chemicals [50, 51]. For example, long exposure of *Aedes aegypti* developing embryo and larvae to lower and higher temperatures (below 3 °C or above 29 °C) of that of their original collection [48, 52] (5–20 °C) was observed to induce female genital appendages to males, and which morphologically appeared to be females [48, 52]. It was also suggested that prolonged exposure of juvenile stages to higher or lower temperatures decreased dimorphism in sex determination, female sex expressivity in heterogametic insects and male sex aberration [47, 51]. Increased females and decreased males relatively to increased egg storage time observed in the present study might have been induced by effects of prolonged egg storage at 4 °C temperature. The temperature (4 °C, 48 ± 2% RH) might have more positive effects on female sexual expressivity than on males.

The findings of the present study can be considered to be of significance in vector and malaria infection control. It is clear that 4 °C, 48 ± 2% RH can be used as the temperature to store *Anopheles* eggs in mass rearing of *An. gambiae* under insectary conditions without significantly affecting adult sex ratio. Since there were higher numbers of emerged males than females from eggs that were stored for 5 days (4 °C, 48 ± 2% RH) compared to eggs stored for 10 and 15 days (4 °C, 48 ± 2% RH), and since sterile insect technique requires only male mosquitoes for sterilization, 5 days at 4 °C and 48 ± 2% RH can be considered the optimal duration and condition for storing *An. gambiae* eggs to be used in mass rearing for sterile insect technique.

Overall, the present study has shown that storing *An. gambiae* eggs for 5 days at 4 °C is an optimal condition for storage with no significant effects on emerged adults sex ratios. This storage condition did not affect *An. gambiae* egg hatch rates, larval development and larval survivorship and, therefore, this condition can be used in storage of anopheline eggs for a period of time to permit flexibility in accommodating the routine or absence of personnel involved in mass rearing of mosquitoes. The study may also be important in establishing a protocol for raising male mosquitoes in sterile insect technique.

## Conclusion

Findings of this present study can be used for information on storage conditions of anopheline eggs as well as the establishment of mass rearing protocols for *An. gambiae* mosquitoes. More research is needed to explore emerged adult survival characteristics, such as mating competitiveness, response to insecticide exposure, vector competence as well as molecular and genetic status of reared adult mosquitoes to determine whether the proposed storage temperature and storage time induced any molecular variations.
